# Maternal myasthenia gravis: a cause for arthrogryposis multiplex congenita

**DOI:** 10.1007/s11832-015-0690-8

**Published:** 2015-10-19

**Authors:** Jana Midelfart Hoff, Anna Midelfart

**Affiliations:** Department of Neurology, Haukeland University Hospital, 5021 Bergen, Norway; Faculty of Medicine, Norwegian University of Science and Technology (NTNU), 7000 Trondheim, Norway

**Keywords:** Myasthenia gravis, AMC, Neonatal myasthenia gravis, Autoimmune disease

## Abstract

**Background:**

Arthrogryposis multiplex congenita (AMC) is a condition defined as contractures in more than two joints and in multiple body areas. The principal mechanism leading to the development of AMC in utero is decreased fetal movement.

**Objective:**

Both fetal and maternal factors can lead to this condition, including maternal myasthenia gravis (MG) which is the topic of this review. MG is an autoimmune disease in which antibodies (immunoglobulin G) are formed against acetylcholine receptors. The disease can affect both genders, but women are more prone to develop the disease in early adulthood, a phase of life when the focus of many women is often directed towards founding a family. During pregnancy, maternal antibodies are transmitted to the fetus.

**Results:**

Although the child is unaffected in most cases, the constant transmission of antibodies in utero can lead to neonatal myasthenia post-partum, a transient condition characterized by hypotonia and swallowing/respiratory difficulties as well as AMC.

**Conclusion:**

The maternal antibody profile in mothers with MG seems to play a key role in whether the child develops AMC or not. There are also indications that there may be a relation between neonatal MG and AMC, as well as a high recurrence rate in siblings.

## Introduction

Arthrogryposis multiplex congenita (AMC) is a condition defined as congenital contractures in more than two joints and in multiple body areas [1]. The condition can occur alone, or it may be associated with multiple developmental defects and be a part of a large number of syndromes with or without central nervous system involvement [1]. The prevalence has been reported to be between 9 and 20 per 100,000 general population [2, 3].

The principal mechanism leading to the development of AMC is decreased fetal movements (fetal akinesia), which can result from a large number of both fetal and maternal disorders [4]. Maternal myasthenia gravis (MG) is one of the conditions that has been linked to the development of fetal AMC [5–7].

## Myasthenia gravis

Myasthenia gravis is a relatively rare neurological disease associated with the formation of antibodies to the acetylcholine receptors (AChR) at the neuromuscular junction, consequently leading to receptor loss [8]. The disease is characterized by fluctuating pathological painless muscle weakness with remissions and exacerbations involving one or several skeletal muscle groups.

The prevalence of MG in the general population has been reported to be about 5–15 per 100,000 [9, 10]. The disease has two peaks, at age 20–40 years and 60–80 years. Whereas the incidence in women and men is equal, women tend to dominate the first peak, men the second [11].

The diagnosis of MG is based on five elements: clinical examination, neurophysiological testing (single-fiber electromyography), pharmacology (Tensilon test: acetylcholine esterase-inhibiting drug), immunology (the detection of AChR antibodies) and thymus pathology (thymus hyperplasia or thymoma).

## MG and pregnancy

There is a two-way relationship between pregnancy and maternal autoimmune disease: pregnancy-induced changes can affect the activity of the disease and the disease can affect the outcome of pregnancy and the child. The main point is that the child is grafted on to the mother, and that immunoglobulin G (IgG) antibodies, such as in MG, can cross the placenta and affect the child both in utero and in the neonatal stage. Due to degradation of the maternally derived IgG, the effect upon the infant will usually be transient, but in some cases the damage caused is irreversible.

## Neonatal MG and AMC

Between 10 and 20 % of infants born to women with MG develop neonatal MG, caused by the maternal IgG antibodies to AChR crossing the placenta [12]. Approximately 80 % of the affected children will develop symptoms during the first 24 h of life, but the condition can develop up to 4 days after birth [12]. The symptoms are usually mild or moderate, including poor sucking and generalized hypotonia, and the condition usually resolves within a few weeks [13]. Respiratory support and tube feeding are only necessary in few cases, but close observation of the newborn of every MG mother is important in order to detect involvement of respiratory or swallowing muscles.

Why neonatal MG develops in only 10–20 % of babies born to MG mothers is still unclear [12]. The antibody epitope specificity of the mother has been suggested to be a major factor. There are two forms of the AChR antibody: one is mainly directed at the fetal AChR, and the other is directed at the adult AChR found in mature endplates [14]. An association between a high ratio of anti-embryonic AChR antibodies has been reported, as well as higher anti-AChR titers in affected versus asymptomatic newborns [15]. The presence of neonatal MG in one sibling seems to be a reliable predictor of a higher risk for the next sibling [16, 17].

The continuous flow of AChR antibodies during pregnancy may also lead to seriously decreased fetal movement and resulting AMC. The severity of AMC in children born to MG mothers is variable and has not been found to correlate with the severity of the mother’s MG, neither at onset time nor during pregnancy [6]. There is, however, a high recurrence rate for giving birth to another child with AMC in these MG mothers.

Regarding neonatal MG, it is not clear why only some MG mothers have affected children. Again, AChR antibody epitope specificity has been proposed as a key factor [18]. An association between neonatal MG and AMC has been reported, where the siblings of an affected child—either with neonatal MG and AMC—have an increased risk for developing either condition [5, 18]. This finding indicates a shared pathophysiology for the two conditions.

Women of fertile age with MG have a lower risk for neonatal MG in their future children if they have a thymectomy, most likely because thymectomy results in reduced antibody load and, thereby, reduced transmission of antibodies across the placenta [19]. Whether this is true for AMC as well needs to be investigated.

## Clinical implications

The pregnant woman with a chronic disease imposes a challenge to medicine. The aim should be a careful balance between treating the mother optimally and preventing harm to the child. This can best be achieved through close cooperation between physicians from different branches of medicine. It is important to discuss pregnancy with female MG patients wanting to have child, as well as to discuss previous pregnancy outcomes in those who have children already, with the aim to optimize treatment. In cases of children with AMC of unknown cause, a thorough genetic work-up is usually performed. In addition to the genetic examination, we recommend examining maternal blood samples for AChR antibodies and referring the patient to a neurologist.
